# Effect of extraocular muscle motion style acupuncture treatment on a patient with oculomotor nerve palsy unresponsive to conventional treatment: A case report

**DOI:** 10.1097/MD.0000000000043279

**Published:** 2025-07-11

**Authors:** Hyukjin Jeong, Nam-Woo Lee, Jinho Lee, Joon-Shik Shin, In-Hyuk Ha, Yoon Jae Lee

**Affiliations:** a Jaseng Hospital of Korean Medicine, Seoul, Republic of Korea; b Jaseng Spine and Joint Research Institute, Jaseng Medical Foundation, Seoul, Republic of Korea.

**Keywords:** case report, diplopia, exotropia, motion style acupuncture, ophthalmoplegia

## Abstract

**Rationale:**

Oculomotor nerve palsy (ONP) is a condition with limited treatment options and significantly affects patients’ quality of life due to its prolonged recovery period, typically lasting 3 to 6 months. This case report describes a patient with ONP, unresponsive to conventional treatment, who was treated with integrative Korean medicine (KM), including extraocular muscle motion-style acupuncture treatment (MSAT).

**Patient concerns:**

A 43-year-old woman presented with symptoms of left ophthalmoplegia, diplopia, and ptosis during postpartum care after undergoing a cesarean section for preeclampsia.

**Diagnoses:**

On the day of onset, magnetic resonance imaging and clinical evaluation confirmed a diagnosis of left third cranial nerve (oculomotor) palsy.

**Interventions:**

She received inpatient treatment followed by outpatient steroid therapy; however, she experienced no improvement for 4 weeks following onset. Subsequently, underwent integrative KM treatment, including extraocular muscle MSAT, over 6 weeks. The MSAT involved the insertion of acupuncture needles into the affected extraocular muscles, followed by guided eye movements in the directions with impaired mobility while the needles remained in place.

**Outcomes:**

After 6 weeks of treatment, the patient’s symptoms, including ptosis, diplopia, and exotropia, improved markedly.

**Lessons:**

This case suggests that integrative KM treatment, particularly extraocular muscle MSAT, may facilitate faster recovery in patients with ONP, highlighting its potential as an effective therapeutic approach for patients unresponsive to conventional treatment. Further validation through large-scale randomized controlled trials is necessary to confirm the efficacy of this method.

## 1. Introduction

The oculomotor nerve, along with the trochlear and abducens nerves, is a major cranial nerve responsible for eyeball and eyelid movement. Patients with oculomotor nerve palsy (ONP) may present with exotropia, characterized by a “down and out” eye deviation.^[1,2]^ Early clinical symptoms of ONP include diplopia, ptosis, and mydriasis. In some cases, patients may also exhibit systemic, ophthalmological, or neurological symptoms.^[3]^ ONP is common in individuals over 60 years old, and its etiology includes microvasculopathy, trauma, tumors, surgery, and aneurysms. Studies have indicated that patients with 2 or more risk factors or intracranial abnormalities on neuroimaging experience longer recovery periods.^[4,5]^ Identifying a definitive cause through clinical evaluation and imaging is the first step in managing ONP. Treatment options include nonsurgical approaches, such as medication, and surgical interventions. Some patients achieve partial or full recovery with conservative treatment; hence, careful observation over at least 6 months is advised. If recovery remains unsatisfactory within 6 months to a year, surgical treatment may be considered.^[1,6,7]^

The recovery rate of patients with ONP varies depending on the underlying condition and etiology. Lee et al conducted a retrospective review of medical records from patients with ONP to examine its clinical features and natural course. The complete recovery rate was 67.5%, with higher rates observed in patients with microvasculopathy and those with minimal extraocular movement limitation at the initial visit. Conversely, patients with trauma or intracranial tumors had lower recovery rates.^[8]^ Recovery times, often spanning 3 to 6 months, can significantly burden patients and affect their quality of life. Consequently, there is growing interest in treatment methods that enhance recovery rates and reduce recovery Times.^[8]^ Traditional Korean medicine (KM) treatments, including acupuncture and moxibustion, have been explored as potential options. Kim et al reported a case of a patient with ONP who achieved complete recovery from ophthalmoparesis, ptosis, and diplopia after 15 weeks of treatment involving acupuncture and moxibustion.^[9]^

Motion style acupuncture treatment (MSAT) combines acupuncture with guided passive or active movement of the patient while the needles remain inserted at specific acupoints. Unlike conventional static acupuncture, MSAT provides additional mechanical stimulation through movement, which is believed to enhance neuromuscular activation and therapeutic outcomes.^[10]^ It has shown effectiveness in managing musculoskeletal disorders and is increasingly used in traditional medicine practices in South Korea and China.^[11]^ According to a scoping review of clinical research on MSAT, Kim et al (2023) reported significant effects in reducing pain and improving functional disability, primarily in patients with musculoskeletal pain.^[11]^ Proposed mechanisms include interrupting the cycle of pain, promoting self-regulation through combined stimulation, and preventing connective tissue adhesion via joint mobilization. However, research on the effectiveness and safety of MSAT remains limited. While MSAT has shown rapid effects in acute pain conditions, its use in neurological disorders such as paralysis typically requires repeated sessions, though few studies have explored this area.^[11]^

Extraocular muscle MSAT involves inserting needles directly into the affected extraocular muscles, followed by eye movement in the directions exhibiting impaired mobility while the needles are retained. While previous studies on KM treatments for ONP have primarily focused on acupuncture, moxibustion, and herbal medicine, no case reports have explored the use of MSAT. This report describes a case in which integrative KM treatment, with MSAT as the primary modality, was applied to a patient with ONP who exhibited symptoms of ophthalmoparesis, diplopia, and ptosis, resulting in improvement across all symptoms. This case report focused on an individual patient and was exempt from approval by our hospital’s Institutional Review Board (JASENG 2024-09-011). Informed consent was obtained from the patient for publication of the details of this case report.

## 2. Case presentation

A 43-year-old woman with no past medical history presented with symptoms of left ophthalmoplegia, diplopia, and ptosis on May 28, 2023, during postpartum care after undergoing a cesarean section for preeclampsia. On the day of onset, she visited Gangnam Severance Hospital in Seoul, and brain magnetic resonance imaging (MRI) revealed no significant abnormalities. Taken together, the MRI and clinical evaluation confirmed a diagnosis of left third cranial nerve (oculomotor) palsy. She received inpatient treatment from June 4 to June 9, 2024, followed by outpatient steroid therapy. The patient was prescribed oral prednisolone with an initial dose of 60 mg daily for 2 days, which was then tapered to 40 mg daily for 7 days and finally reduced to 20 mg daily for 11 days. Additionally, aspirin at a dose of 100 mg daily, rosuvastatin calcium at 20.8 mg daily, and rebamipide at 150 mg twice daily were administered, all for a duration of 21 days. Esomeprazole magnesium trihydrate at a dose of 44.5 mg daily for 21 days was also included in the regimen to manage potential gastrointestinal side effects.

**Figure 1.**
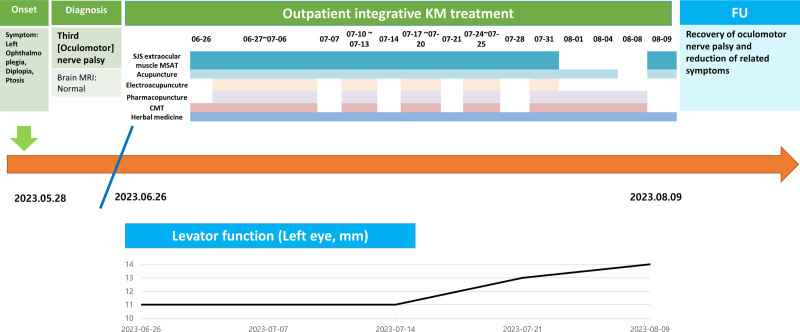
Outline of interventions and trend in functional recovery.

Nevertheless, the patient’s symptoms persisted, and she visited our hospital on June 26, 2023, 1 month after symptom onset. The patient underwent 27 sessions of integrative KM treatment between June 26 to August 9, 2023. The treatment included 24 sessions of extraocular muscle MSAT, 26 sessions of acupuncture, 18 sessions each of electroacupuncture and pharmacopuncture, 19 applications of pharmacopuncture eye drops, 22 sessions of Chuna manual therapy, and herbal medicine prescriptions (Fig. 1).

MSAT = motion-style acupuncture treatment, CMT = Chuna manual therapy.

**Figure 2.**
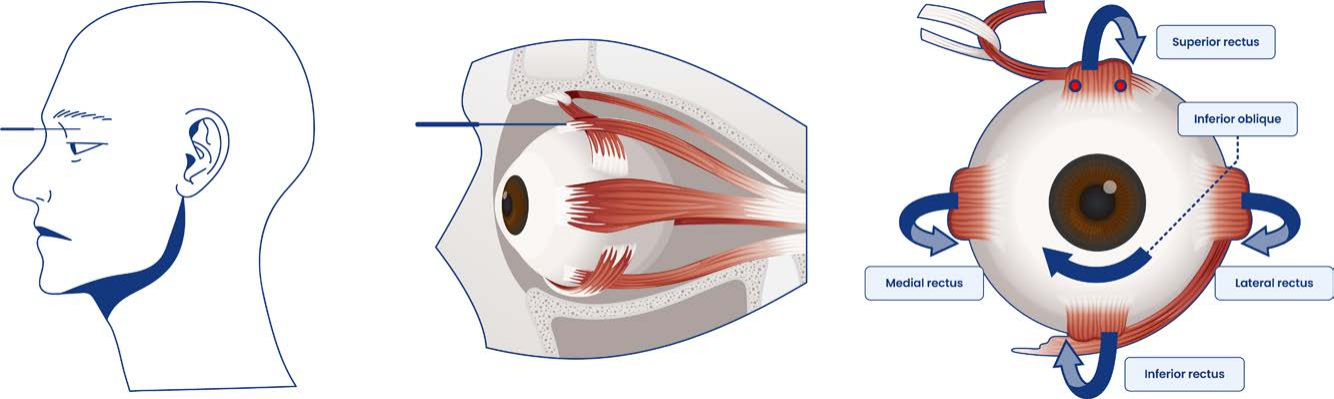
Application of extraocular muscle MSAT. MSAT = motion-style acupuncture treatment.

Extraocular muscle MSAT was performed by a Korean medicine doctor (KMD). The patient was seated with her lower back, upper back, and neck straightened, and her chin slightly tucked. Disposable stainless-steel filiform needles (30 mm × 40 mm) were inserted into the superior rectus muscle. During needle retention, the KMD placed a finger in front of the patient’s eye and instructed her to move the eyeball in all directions, including the direction of impaired mobility, for approximately 10 minutes to promote normal eye movement (Fig. 2).

Along with extraocular muscle MSAT, the KMD administered acupuncture using disposable stainless-steel filiform needles (30 × 40 mm). Acupuncture was performed at the patient’s affected-side acupoints, including Shangguan (GB3), Wangu (GB12), Fengchi (GB20), Yifeng (TE17), Cuanzhu (BL2), Sizhukong (TE23), Chengqi (ST1), and Jingming (BL1), as well as the unaffected-side Cuanzhu (BL2), Sizhukong (TE23), and Jingming (BL1). The anatomical locations of these points are provided in Content S1, Supplemental Digital Content, https://links.lww.com/MD/P377. The needles were retained for 15 minutes without any additional manual stimulation such as rotation or lift-thrust techniques.

Electroacupuncture was concurrently administered during 18 of the total treatment sessions, using CellMac STM-111 device. Electroacupuncture was applied to the affected-side periocular points, including Jingming (BL2) and Cuanzhu (BL2), at a frequency of 2 to 8 Hz for 15 minutes. In sessions where both manual acupuncture and electroacupuncture were performed, they were applied simultaneously after the pharmacopuncture treatment.

Pharmacopuncture therapy involved the injection of 1 mL of Hominis Placenta pharmacopuncture solution (prepared at the extramural herbal dispensary of Jaseng Hospital of Korean Medicine, Seongnam-si, Republic of Korea) into the patient’s affected-side Yifeng (TE17) and Wangu (GB12) using disposable syringes (Kovax-Syringe, 1 mL, KOVAC-MED Co., Ltd., Ansan, Korea). Additionally, Hwangryunhaedok-tang pharmacopuncture solution, prepared at the same dispensary, was instilled as eye drops into the affected eye using the same type of disposable syringe. In sessions where all 3 modalities (manual acupuncture, electroacupuncture, and pharmacopuncture) were applied, the pharmacopuncture injection and eye drop administration were performed first, followed by concurrent manual acupuncture and electroacupuncture.

For Chuna manual therapy, the KMD selected and performed appropriate Chuna techniques, such as craniosacral therapy and nonresistant techniques. Regarding herbal medicine, the patient was prescribed Nokyonggunbi-tang during the first visit and consumed 100 cc in the morning and evening for 25 days. Subsequently, Gamijihwang-tang was prescribed and taken as 100 cc doses each morning and evening for 12 days. Finally, Gobonhwangjeong-hwan was prescribed, with 1 pill taken in the morning and evening for 7 days. The composition of the individual prescriptions is detailed in Content S2, Supplemental Digital Content, https://links.lww.com/MD/P378.

**Figure 3.**
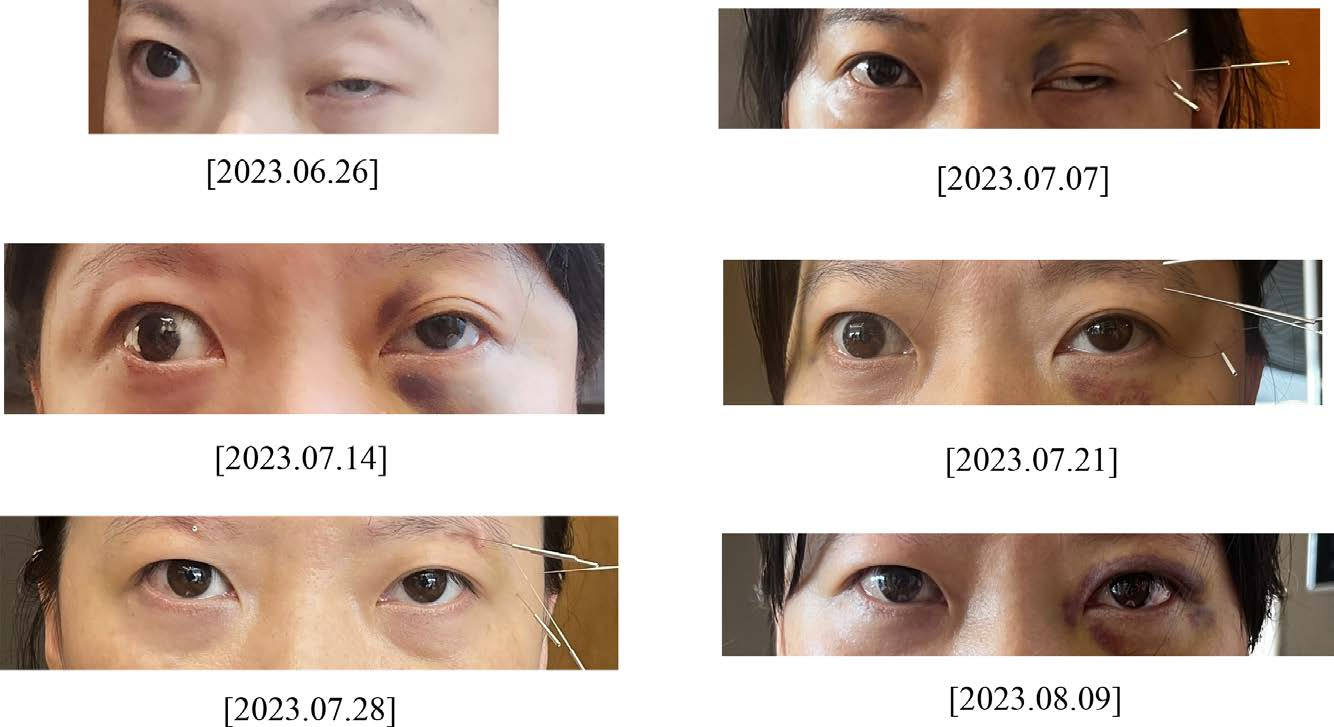
Visible changes in the patient’s left eye over time.

To monitor changes in the ptosis over time, photographs were taken on 6 occasions: the initial visit (June 26, 2023) and subsequently on July 7, 14, 21, 28, and August 9, 2023 (Fig. 3). The levator function (LF) was evaluated to assess improvement in the ptosis. The LF test measured the excursion distance of the upper eyelid margin from maximum downgaze to upgaze while ensuring the frontalis muscle remained immobilized by pressing firmly on both eyebrows with the thumbs. Normal LF is defined as 14 mm or greater, with smaller values indicating ptosis.

On June 26, 2023, the LF test results were 14 and 11 mm on the right and left eyelids, respectively. Other findings included left ophthalmoparesis, horizontal diplopia, a larger left pupil compared to the right, and a diminished left pupillary reflex were noted. On July 7, 2023, the measured right and left LF was 14 and 11 mm, respectively, but a slight improvement in the left pupillary reflex was observed. On July 14, 2023, although the measured right and left LF remained 14 and 11 mm, respectively, the left pupillary reflex showed further improvement, and better left medial rectus movements were noted during eye movements. On July 21, 2023, the measured right and left LF was 14 and 13 mm, respectively. Normal convergence and ocular alignment were observed at a distance of 30 cm, with no diplopia reported. On July 28, 2023, the measured right and left LF remained 14 and 13 mm, respectively. The patient reported seeing a single image in a fixed gaze, although 2 images were usually observed, and her diplopia worsened with downward gaze. Finally, on August 9, 2023, the LF was 14 mm bilaterally. The patient demonstrated no loss of focus even at distances >30 cm, no diplopia, and an almost normal range of movement in the left medial rectus during eye movements.

## 3. Discussion

The oculomotor nerve originates from the oculomotor nuclei in the midbrain and comprises motor and autonomic parasympathetic nerve fibers. The motor nerve fibers innervate the superior rectus, inferior rectus, medial rectus, inferior oblique, and levator palpebrae superioris muscles, which control eye and eyelid movements.^[12]^ The autonomic parasympathetic nerve fibers innervate the sphincter pupillae, which constrict the pupil to protect the retina from excessive light exposure. Additionally, these fibers innervate the ciliary muscles, altering the lens curvature to enable focus on target objects.^[12]^ When ONP occurs, 4 extraocular muscles become paralyzed, excluding the superior oblique and lateral rectus muscles. Consequently, the patient exhibits abnormal ocular movements – adduction, depression, and elevation – during eye movement tests, along with exotropia characterized by the “down and out” positioning of the affected eye due to the unopposed actions of the superior oblique and lateral rectus muscles. Clinical signs of ONP include other ocular abnormalities such as ptosis, diplopia, and mydriasis.^[1,12]^ Diagnosis and treatment of ONP vary based on patient age, the type of paresis or paralysis, and the presence of associated symptoms and signs. Treatment priorities are determined by the underlying etiology and the anatomical location or characteristics of the lesion.^[2,6]^ Diplopia is commonly managed by covering the affected eye with an eye patch or using prism glasses. Conservative treatment typically involves medications such as adrenocortical steroids or diuretics to address systemic risk factors and alleviate symptoms.^[1,6]^ However, if ONP does not resolve within 6 months to 1 year, surgical interventions such as medial rectus resection, lateral rectus muscle transposition, or superior oblique muscle transposition are considered. Success in surgical treatment depends on aligning patient expectations with surgical outcomes.^[1,6,13–15]^

Fang et al retrospectively reviewed patients diagnosed with acquired third nerve palsy in Olmsted County, Minnesota, from January 1, 1978, to December 31, 2014. They found a higher annual incidence of acquired ONP in individuals older than 60 years, with the most common causes being microvascular (42%), followed by trauma (12%), neoplasm (11%), neurosurgery (10%), and aneurysms (6%).^[4]^ In line with these findings, the present case was also presumed to involve a microvascular origin, based on the patient’s recent history of preeclampsia and the absence of MRI abnormalities. To address potential vascular involvement, low-dose aspirin and rosuvastatin were administered during the initial 21 days following onset, prior to the start of integrative KM treatment. Similarly, Jung et al reported that among 54 Korean patients diagnosed with the third, fourth, or sixth cranial nerve palsies, ONP accounted for 29.6%, showing a smaller percentage compared to the fourth (35.2%) and sixth (35.2%) nerve palsies. Patients with 2 or more risk factors and intracranial abnormalities detected through neuroimaging experienced prolonged recovery times.^[5]^ Yoon et al observed that the recovery rate for vascular paralytic strabismus was 60.9%, compared to 63.8% for idiopathic paralytic strabismus. Recovery periods averaged 130.1 ± 145.1 days (19–633 days) for vascular cases and 92.6 ± 76.6 days (16–271 days) for idiopathic cases, indicating a typical recovery time of 3 to 6 months.^[16]^ In this case report, improvement began approximately 1.5 months after initiating treatment, with complete recovery achieved in 2.5 months, representing a notably rapid recovery. Integrative KM treatment, including extraocular muscle MSAT and acupuncture, likely contributed to this accelerated outcome. A similar timeline of improvement was observed in a previous case report by Nurwati et al (2023), in which acupuncture led to the resolution of diplopia and ptosis following subdural hematoma surgery.^[17]^ However, unlike the present case, that report involved conventional acupuncture without the application of MSAT or integrative KM modalities. This finding is consistent with existing literature suggesting that acupuncture facilitates faster recovery in patients with oculomotor nerve dysfunction. Although research on acupuncture’s efficacy and mechanisms for ONP remains limited, existing studies suggests that acupuncture facilitates faster recovery, enhances quality of life, and helps manage comorbid conditions such as hypertension and diabetes mellitus.^[18,19]^ A systematic review of 18 randomized controlled trials involving 1150 patients concluded that acupuncture may surpass conventional treatments in clinical efficiency, diplopia scores, palpebral fissure size, pupil diameter, and quality of life metrics.^[20]^ Yang et al highlighted that acupuncture and electroacupuncture are effective for nerve regeneration, promoting recovery by upregulating nerve growth factors (e.g., nerve growth factor, neurotrophin-3, brain-derived neurotrophic factor, and glial cell line-derived neurotrophic factor) and activating the brain-derived neurotrophic factor-Tropomyosin receptor kinase B signaling pathway. Acupuncture further enhances nerve function recovery by increasing Annexin A5 and collapsin response mediator protein 2 protein expression, downregulating glial fibrillary acidic protein and chondroitin sulfate proteoglycans, and reducing neuronal cytoplasmic edema via the Wingless/Integrated signaling pathway, which plays a key role in regulating neural cell survival, axonal regeneration, and synaptic plasticity.^[21]^

The effects of acupuncture can be enhanced by additional stimulation through movement following needle insertion.^[11]^ MSAT is commonly used in KM clinical practice in South Korea to alleviate musculoskeletal pain and is gradually gaining popularity in China.^[11]^ Although the exact mechanism of MSAT remains unclear, its combined effects on pain, such as breaking the negative cycle of pain and the role of joint movement in preventing tissue adhesion, have been widely discussed in the literature.^[11]^ Previous studies have used acupoints in the periocular region, but none have employed direct needling into the extraocular muscles.^[20]^ In this study, needle insertion into the extraocular muscles directly stimulated the paralyzed muscles, and eye movement was performed while the needles were retained at the acupoints. This approach promotes nerve function recovery by applying acupuncture stimulation to paralyzed muscles and providing additional stimulation through movement by inducing eye movement in directions where mobility is restricted.^[11,21]^ This treatment modality can effectively activate paralyzed muscles and nerves, promote functional recovery, and offer a differentiated approach by directly stimulating the paralyzed muscles, in contrast to conventional acupuncture methods.

While our report demonstrated an unusually fast recovery from ONP that may be attributable to the integrative KM treatment, including MSAT, this report includes only 1 case, and further research is needed to confirm the efficacy of MSAT for ONP. Moreover, as the intervention in this case report was an integrated KM treatment involving multiple methods, such as acupuncture, pharmacopuncture, Chuna manual therapy, and MSAT, further investigation is necessary to isolate the specific contribution of MSAT. Another consideration is that because the extraocular muscle is a sensitive area directly connected to the eye, careful attention and special care are required during the procedure. Extraocular muscle MSAT is thus limited by its technically challenging nature, and safe administration can only be ensured when performed by skilled specialists. In this case report, the patient developed a hematoma in the periocular area following acupuncture therapy at the Seungeup (ST1) and Jeongmyeong (BL1) acupoints. Specifically, hematomas occurred near the BL1 point during the 8th and 23rd treatment sessions and near the ST1 point during the 12th session, out of a total of 27 sessions. These points are located in the periocular area, which contains numerous microvessels, making them more susceptible to microbleeds or hematomas than other areas. However, the hematoma observed in this case were not severe. Following each occurrence, treatment around the affected area was minimized, and the hematomas improved naturally, similar to the typical recovery process after general acupuncture therapy.

In summary, the findings of this case report suggest that MSAT may have a positive impact on recovery from ONP by promoting movement. If MSAT is implemented early in the treatment process, it could contribute significantly to faster recovery. However, to establish the efficacy and safety of MSAT with high-quality evidence, large-scale randomized controlled trials and observational studies with larger sample sizes are necessary. Further research will provide scientific evidence supporting the efficacy of MSAT for ONP, enabling its applicability and safety to be assessed across different patient populations.

## Author contributions

**Conceptualization:** Nam-Woo Lee, Jinho Lee, Yoon Jae Lee.

**Methodology:** Nam-Woo Lee, Jinho Lee.

**Supervision:** Joon-Shik Shin, In-Hyuk Ha, Yoon Jae Lee.

**Writing – original draft:** Hyukjin Jeong.

## Supplementary Material


