# Supplementary material for: Effect of extraocular muscle motion style acupuncture treatment on a patient with oculomotor nerve palsy unresponsive to conventional treatment: A case report
Source: Medicine (Baltimore). 2025 Jul 11;104(28):e43279. doi: 10.1097/MD.0000000000043279 (PMC12263013; doi:10.1097/MD.0000000000043279)
Supplement: Supplementary file 1 [file medi-104-e43279-s001.docx]

**Supplemental Digital Content 1. Locations of Acupuncture Points Used in the Treatment**

**
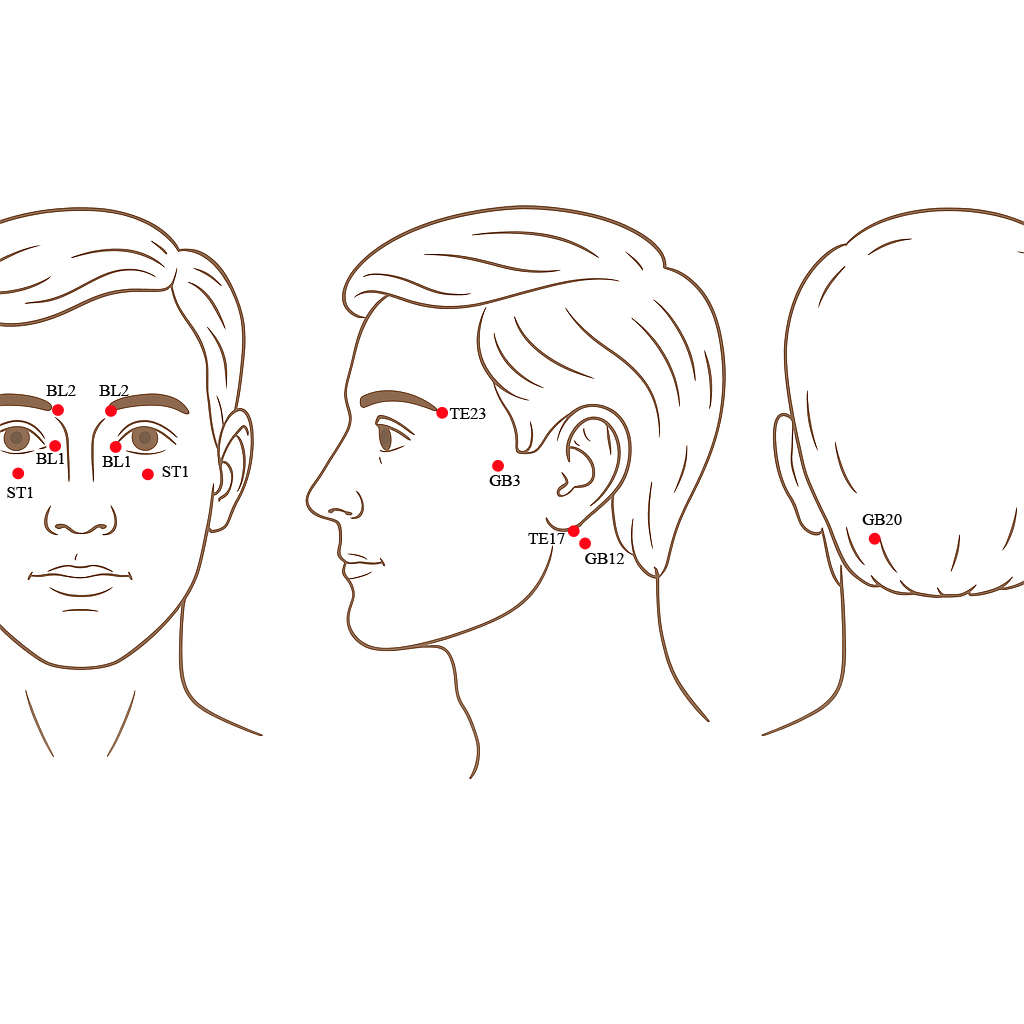
**

Shangguan (GB3): On the head, in the depression superior to the midpoint of the zygomatic arch

Wangu (GB12): In the anterior region of the neck, in the depression posteroinferior to the mastoid

process.

Fengchi (GB20): In the anterior region of the neck, inferior to the occipital bone, in the depression

between the origins of sternocleidomastoid and the trapezius muscles.

Yifeng (TE17): In the anterior region of the neck, posterior to the ear lobe, in the depression anterior to the inferior end of the mastoid process.

Cuanzhu (BL2): On the head, in the depression at the medial end of the eyebrow.

Sizhukong (TE23): On the head, in the depression at the lateral end of the eyebrow.

Chengqi (ST1): On the face, between the eyeball and the infraorbital margin, directly inferior to the

pupil.

Jingming (BL1): On the face, in the depression between the superomedial parts of the inner canthus of

the eye and the medial wall of the orbit.
