# Supplementary material for: Effect of extraocular muscle motion style acupuncture treatment on a patient with oculomotor nerve palsy unresponsive to conventional treatment: A case report
Source: Medicine (Baltimore). 2025 Jul 11;104(28):e43279. doi: 10.1097/MD.0000000000043279 (PMC12263013; doi:10.1097/MD.0000000000043279)
Supplement: Supplementary file 2 [file medi-104-e43279-s002.docx]

| **Supplemental Digital Content 2. Composition of Herbal Decoctions** |  |
| --- | --- |

| Prescription | Composition of Herbs (Scientific Names) |
| --- | --- |
| Lurong Jianpi Tang (鹿茸健脾湯) | - ***Astragalus membranaceus*** (黃芪) - ***Lycium chinense*** or ***Lycium barbarum*** (枸杞子) - ***Angelica gigas*** (當歸) - ***Atractylodes macrocephala*** (白朮) - ***Dimocarpus longan*** (龍眼肉) - ***Ligusticum chuanxiong*** (川芎) - ***Glycyrrhiza uralensis*** (甘草) - ***Cervus nippon*** or ***Cervus elaphus*** (鹿茸粉骨) - ***Saussurea costus*** (木香) - ***Poria cocos*** (茯苓) - ***Amomum villosum*** (砂仁) - ***Crataegus pinnatifida*** (山楂) - ***Rehmannia glutinosa*** (熟地黃) - ***Panax ginseng*** (人蔘) - ***Paeonia lactiflora*** (芍藥) - ***Fallopia multiflora*** (何首烏) - ***Carthamus tinctorius*** (紅花) |
| Jiawei Dihuang Tang (加味地黃湯) | - ***Rehmannia glutinosa*** (熟地黃) - ***Angelica gigas*** (當歸) - ***Cornus officinalis*** (山茱萸) - ***Dioscorea opposita*** or ***Dioscorea polystachya*** (山藥) - ***Lycium chinense*** or ***Lycium barbarum*** (枸杞子) - ***Chrysanthemum indicum*** (菊花) - ***Cuscuta chinensis*** (菟絲子) - ***Cervus nippon*** or ***Cervus elaphus*** (鹿茸粉骨) - ***Paeonia suffruticosa*** (牡丹皮) - ***Poria cocos*** (茯苓) - ***Ligusticum chuanxiong*** (川芎) - ***Alisma orientale*** (澤瀉) |
| Guben Huanjing Wan (古本還睛丸) | - ***Ophiopogon japonicus*** (麥門冬) - ***Rehmannia glutinosa*** (熟地黃) - ***Rehmannia glutinosa*** (地黃) - ***Asparagus cochinchinensis*** (天門冬) - ***Lycium chinense*** or ***Lycium barbarum*** (枸杞子) - ***Poria cocos*** (茯苓) - ***Dioscorea opposita*** or ***Dioscorea polystachya*** (山藥) - ***Panax ginseng*** (人蔘) - ***Cassia obtusifoliaa*** (決明子) - ***Chrysanthemum indicum*** (菊花) - ***Dendrobium nobile*** (石斛) - ***Achyranthes bidentata*** (牛膝) - ***Citrus aurantium*** (枳殼) - ***Cuscuta chinensis*** (菟絲子) - ***Prunus armeniaca*** (杏仁) - ***Saposhnikovia divaricata*** (防風) - ***Saiga tatarica*** (羚羊角) - ***Celosia argentea*** (靑葙子) - ***Glycyrrhiza uralensis*** (甘草) - ***Schisandra chinensis*** (五味子) - ***Tribulus terrestris*** (蒺藜子) - ***Ligusticum chuanxiong*** (川芎) - ***Coptis chinensis*** (黃連) |
